# Spatial transcriptomics in cancer research: insights into tumorigenesis, diagnosis and therapeutics

**DOI:** 10.1038/s41420-026-03092-0

**Published:** 2026-06-30

**Authors:** Qiang Wen, Qing Lan, Jianjian Yin, Fei Guo, Xiaosong Wei, Caiyun Wu, Lei Jin, Xu D. Zhang, Jinyan Li, Lirong Zhang, Tao Liu

**Affiliations:** 1 Institute of Clinical Pharmacology, School of Basic Medical Sciences, Zhengzhou Key Laboratory of Lipid Metabolism and Precision Diagnosis & Treatment in Oncology, State Key Laboratory of Metabolic Dysregulation & Prevention and Treatment of Esophageal Cancer, Tianjian Laboratory of Advanced Biomedical Sciences, School of Convergence Medicine, Zhengzhou University, Zhengzhou, China; 2 Department of Neurosurgery, The Second Affiliated Hospital of Soochow University, Suzhou, China; 3 Henan Provincial People’s Hospital, Henan University, Zhengzhou, China; 4 State Key Laboratory of Metabolic Dysregulation & Prevention and Treatment of Esophageal Cancer, Tianjian Laboratory of Advanced Biomedical Sciences, School of Convergence Medicine, Zhengzhou University, Zhengzhou, China; 5 The First Affiliated Hospital of Zhengzhou University, Zhengzhou, China; 6 School of Medicine and Public Health, University of Newcastle, Callaghan, NSW Australia; 7 Translational Research Institute of Henan Provincial People’s Hospital, Henan International Joint Laboratory of Non-coding RNA and Metabolism in Cancer, Zhengzhou, China; 8 Faculty of Computer Science and Artificial Intelligence, Shenzhen University of Advanced Technology, Shenzhen, Guangdong, China; 9 Department of Pharmacology, School of Basic Medical Sciences, Zhengzhou University, Zhengzhou, China; 10 School of Clinical Medicine, University of New South Wales, Sydney, NSW Australia

**Keywords:** Cancer genomics, Oncogenes

## Abstract

Spatial transcriptomics is an innovative technology that enables high-throughput, genome-wide analysis of transcript expression and spatial localization within tissues. By preserving structural organization, it provides critical insights into tumor sub-regions, substructures, and the heterogeneity and plasticity of cancer, stromal, and immune cells, as well as cell–cell interactions. Spatial transcriptomics also offers an unprecedented understanding of the tumor microenvironment, including immune cell infiltration, activation and repression, and immune suppression mediated by stromal cells. Importantly, it deepens our understanding of malignant transformation from precancerous lesions, tumorigenesis, and immune escape. Furthermore, spatial transcriptomics is reshaping cancer subtype diagnosis and risk stratification, uncovering factors associated with drug resistance, predicting therapy responses, and informing the development of personalized cancer treatments and potentially prevention. In summary, spatial transcriptomics serves as a cornerstone of cancer research, transforming the research landscape and unlocking groundbreaking opportunities for precision cancer diagnosis, risk stratification, targeted therapy, and prevention.

## Facts

- Spatial transcriptomics is an innovative technology that enables high-throughput genome-wide analysis of the expression and spatial localization of transcripts within tissues.
- Spatial transcriptomics enables critical insights into tumor subregions, complex cellular components and cell-cell interactions.
- Spatial transcriptomics offers unprecedented comprehension of the tumor microenvironment, immune escape, malignant transformation, tumor initiation and progression.
- Spatial transcriptomics remodels cancer diagnosis and risk stratification, uncovers novel mechanisms of drug resistance, predicts therapy response and informs treatment decision-making.

## Open questions

- How to better understand cell-cell interactions with spatial transcriptomics?
- How to effectively integrate spatial transcriptomics with multi-omics, such as proteomics, epigenomics, and metabolomics?
- How to combine spatial transcriptomics with artificial intelligence and machine learning to identify novel biomarkers for prognosis, risk stratification, and therapeutic response prediction?

## Introduction

As RNA sequencing (RNA-seq) data generated from bulk cells or tissues represent the average gene expression in many cells, single-cell RNA-seq was developed. Single-cell RNA-seq technology has led to a number of paradigm-shifting discoveries, due to its ability to reveal cell type composition of bulk cells and tissues as well as heterogeneity and complexity of RNA transcripts in different cell types [1, 2].

Spatial transcriptomics has further revolutionized our understanding of the gene expression by providing a spatially resolved perspective within complex tissues. While single-cell RNA-seq data do not provide spatial information of dissociated cells, spatial transcriptomics can investigate gene expression in cells from tissues with spatial information through spatial barcodes [3, 4], in situ sequencing [5] and imaging-based single-molecule fluorescence in situ hybridization (smFISH) [6]. Gene expression from one to tens of cells can be investigated in 5000 spots on a single tissue slide, and spatial transcriptomics makes it possible to identify differential gene expression in cells under various local microenvironment within tissues.

Spatial barcoding platforms, such as 10× Genomics Visium, use fresh-frozen or paraffin-embedded tissue sections and capture transcriptional profiles from multiple cells per spot. This makes them suitable for identifying regional expression patterns but limits single-cell or subcellular resolution [3]. The spot-size limitation means that each measurement represents an average of several cells, which can mask rare cell populations or dilute signals from small but biologically important niches [7]. In comparison, in situ sequencing methods (e.g., CosMx) and smFISH technologies (e.g., MERFISH) achieve subcellular resolution by imaging individual RNA molecules, but require optimized tissue fixation and multi-round hybridization/imaging, making them more sensitive to tissue quality [5, 6]. Batch effects arise from differences in tissue handling, slide chemistry, capture efficiency, imaging conditions, and platform-specific biases. Standardized tissue processing and experimental design, platform-specific normalization and computational integration tools are required to address them [8].

Effective integration of spatial transcriptomics with complementary multi-omics datasets involves cellular deconvolution, where single-cell or single-nucleus RNA-seq atlases are used as a reference to infer-cell transcriptional states and cell-type composition within each lower-resolution spatial transcriptomics spot. Tools like SPOTlight [7] and RCTD [9] employ statistical models to map single-cell RNA-seq-derived cell types onto spatial coordinates, thereby “enhancing” spatial transcriptomics data with single-cell resolution. For broader molecular profiling, spatial transcriptomics data can be correlated with region-matched bulk RNA-seq using integration frameworks, such as the Spatial Transcriptomics Graph Attention Network [10], and spatially resolved RNA and protein co-expression in tissues can be analyzed by techniques, such as multiplex digital spatial profiling [11], spatial multi-omics platforms [12] and spatial protein and transcriptome sequencing [13].

The integration of high-resolution imaging data adds crucial morphological context. Tools like STalign or PASTE can be used to align multiple spatial transcriptomics sections [14, 15], while tools, like LETSmix, deconvolve cell types through integrating spatial correlations to refine spatial transcriptomics data [16]. Analytical frameworks, such as Squidpy [17], Open-ST [18], and Giotto [19], integrate spatial transcriptomics and imaging data and enable unified neighborhood analysis, allowing researchers to model cell-cell communication by jointly analyzing ligand-receptor expression from spatial transcriptomics and receptor protein localization from imaging. Together, these strategies transform spatial transcriptomics into a central hub for building causal, multi-scale models of tissue function.

In this review, we explore the key applications of spatial transcriptomics in cancer research, including the identification of tumorigenic factors; a deeper understanding of tumor initiation, progression, diagnosis, and risk stratification; the identification of candidate transcripts and pathways associated with drug resistance; and the prediction of effective therapies and prevention for individual patients.

## Spatial transcriptomics reveals tumor subregions, complex cellular components and cell–cell interactions

**Fig. 1:**
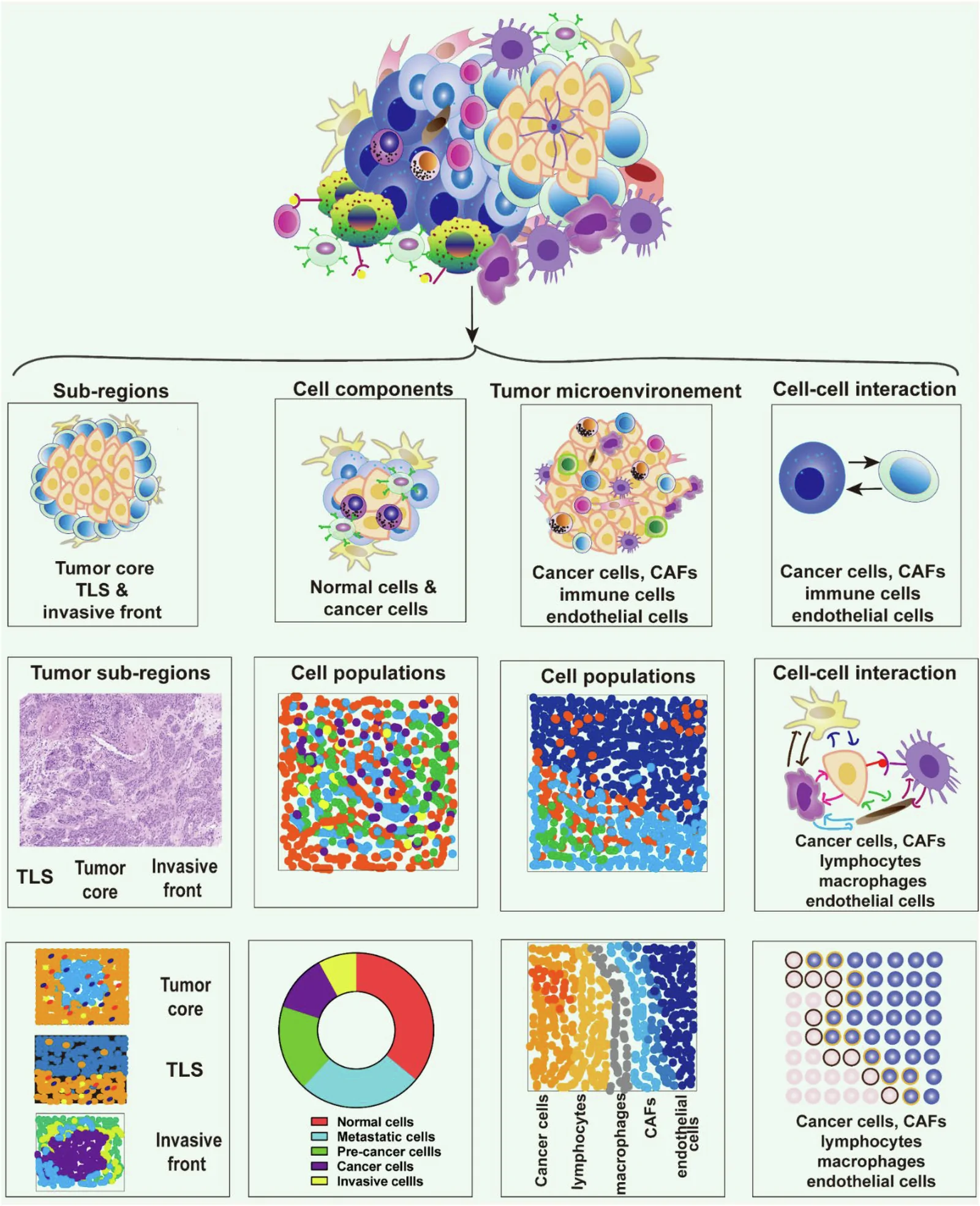
Overview of spatial transcriptomics analysis in cancer research. Spatial transcriptomics technology has been applied to: (i) examine tumor sub-regions, such as the tumor core, invasive front, and tertiary lymphoid structures (TLSs); (ii) analyze cellular components involved in tumor initiation (the transition from pre-cancerous to cancerous cells), progression (cancer cells), and metastasis (invasive and metastatic cancer cells); (iii) facilitate research on the tumor microenvironment, including immune cells (lymphocytes and macrophages), cancer-associated fibroblasts (CAFs), endothelial cells, and responses to immunotherapy; and (iv) investigate cell–cell interactions critical for tumor initiation, progression, metastasis, and treatment response.

Very briefly, spatial transcriptomics has revolutionized our ability to: (i) unravel tumor sub-regions, such as the tumor core, invasive front, and tertiary lymphoid structures (TLSs); (ii) analyze complex cellular components, including normal, pre-cancerous, cancerous, invasive, and metastatic cells; (iii) investigate the tumor microenvironment (TME), which includes cancer cells, cancer-associated fibroblasts (CAFs), lymphocytes, macrophages, and endothelial cells; and (iv) identify and characterize cell–cell interactions critical to tumor initiation, progression, metastasis, and treatment response (Fig. 1).

Pan-cancer studies of 15 cancer types have defined a set of gene signatures that characterize various cancer cell states, including cell identity, cell cycle, interferon response, stress response, hypoxia, epithelial-to-mesenchymal transition (EMT), and metal response [20]. Spatial transcriptomics analyses of tumors also reveal consistent colocalizations of cancer cell states with specific cell types in the TME, such as the association between the interferon response gene signature in cancer cells and macrophages and T cells, and the positive correlation between cancer cells undergoing EMT and CAFs, suggesting cell–cell interactions [20].

## Spatial transcriptomics highlights the critical roles of the complex tumor microenvironment and its constituent cells

### Tumor microenvironment

**Fig. 2:**
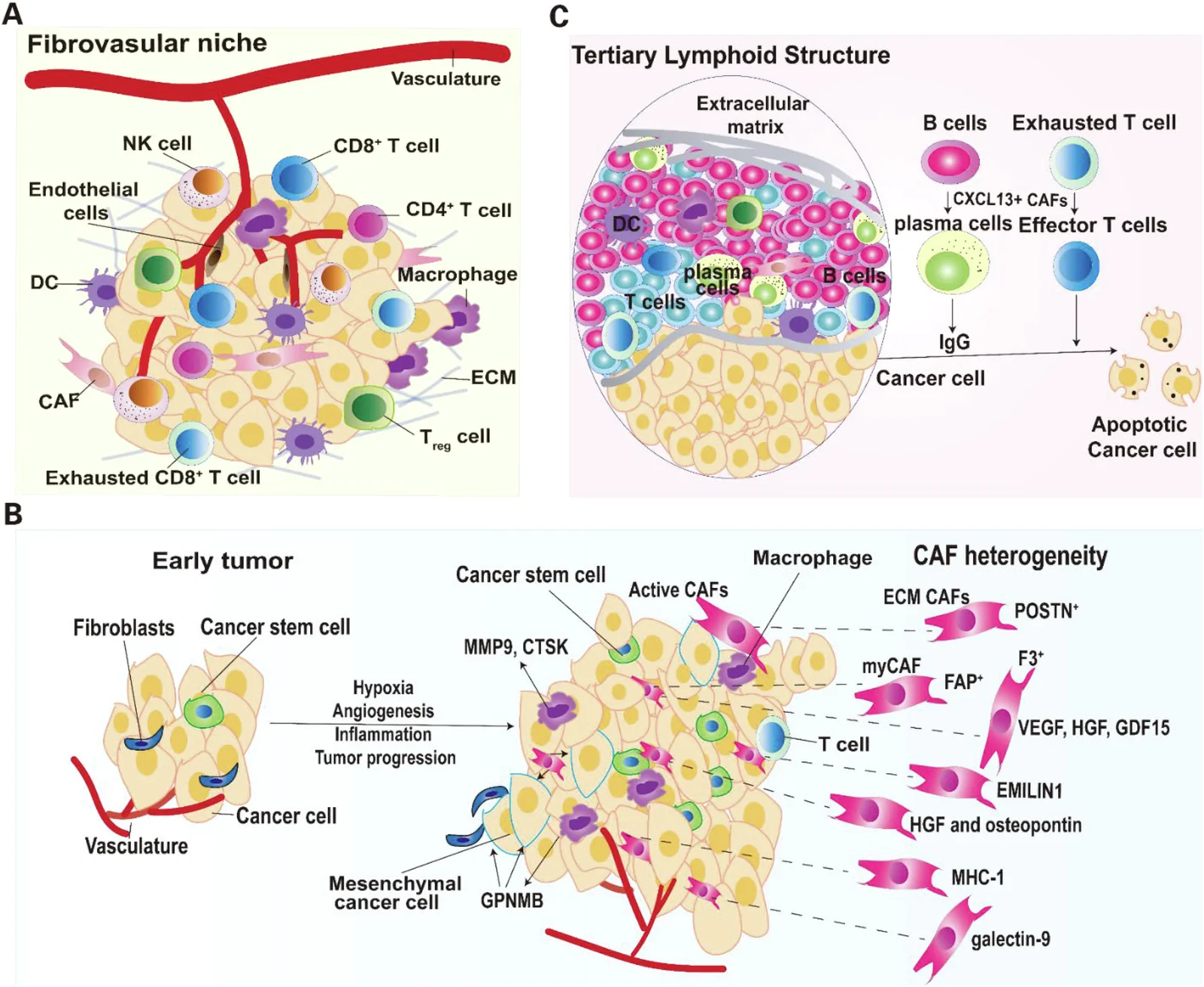
Spatial transcriptomics data highlight the critical roles of the complex tumor microenvironment, its constituent cells, and substructures. **A** The tumor microenvironment comprises cancer cells, immune cells, including macrophages and various lymphocytes, such as T cells, NK cells, and dendritic cells (DCs), cancer-associated fibroblasts (CAFs), endothelial cells, and the extracellular matrix (ECM). **B** During tumor development, fibroblasts evolve into CAFs, and different subpopulations of CAFs interact with cancer stem cells, cancer cells, and immune cells, leading to cancer stem cell enrichment, cancer cell mesenchymal transition, CD8^+^ T cell exhaustion, immune suppression, tumor progression, evasion, metastasis, and resistance to therapies. **C** Tertiary lymphoid structures (TLSs) are associated with T cell transition to effector T cells, B cell maturation, differentiation into IgG-producing plasma cells, enhanced immune responses, and cancer cell apoptosis.

10× Genomics Visium spatial transcriptomics analysis of tumors from 78 patients with six different cancer types reveals that tumor micro-regions can be summarized as cancer cell clusters separated by stroma, and that spatial sub-clones with differential genetic mutations or copy number variations exhibit varying oncogenic activities [21]. Augmented immune exhaustion markers and immune cold and hot neighborhoods are found in the subclones, with macrophages primarily present at tumor boundaries [21] (Fig. 2A).

Integrated Nanostring GeoMx or 10× Genomics Visium, both barcoding-based spatial technologies, with single-cell transcriptomics analyses of human colorectal and gastric cancer tissues, uncover tumor subregions enriched with T cells, myeloid cells that express high levels of T cell-attracting chemokines, cancer cells that interact with T cells and myeloid cells, and cancer stem cells that interact with immunosuppressive M2 macrophages and CXCL13⁺ T cells to evade immune surveillance [22, 23] (Fig. 2A). 10× Genomics Visium analyses of non-small cell lung squamous cell carcinoma and adenocarcinoma tissues unveil transcriptional oncofetal reprogramming in macrophages, a negative correlation between macrophages and NK cells/T cells in both types of tumors, as well as considerably different expression levels of immune checkpoint inhibitors [24]. The results suggest that the two spatial transcriptomics platforms generate consistent data on immune suppression in both cancer types.

In prostate cancer tissues, 10× Genomics Visium spatial transcriptomics analysis shows that the immunosuppressive TME is characterized by exhausted T cells, a reduction in CD8^+^CXCR6^+^ T cells, upregulation of SOX9^high^AR^low^ cancer stem cells, suppressive myeloid cells, as well as Treg cell infiltration that is possibly caused by FAP^+^ fibroblasts [25, 26]. Single-cell RNA-seq and 10× Genomics Visium profiling of intrahepatic cholangiocarcinoma tissues from the tumor core, invasive front, and adjacent normal tissues reveal that tumor cells at the invasive front are linked with POSTN^+^/FAP^+^ CAFs, endothelial cells, CD8^+^ T cell exhaustion, and CD8^+^ T cell interactions with SPP1^+^ tumor-associated macrophages (TAMs), promoting tumor progression [27]. High-plex imaging and NanoString GeoMx spatial profiling of melanomas identify immune suppression even in precursor regions. Additionally, programmed cell death protein 1 (PD1)/programmed death-ligand 1 (PD-L1) promote interactions among macrophages, dendritic cells (DCs) and T-cells with melanoma cells in invasive tumors, and cytotoxic T cells interact with melanoma cells at tumor regression subregions [28]. The results also suggest that the different spatial transcriptomics platforms generate consistent data on immune suppression in different cancer types.

In summary, spatial transcriptomics is crucial for understanding the TME, as it enables the precise mapping of gene expression patterns in various cell subpopulations within subregions of tumors and helps reveal how different cell subtypes interact within the tumor, providing invaluable insights into the TME and immune responses.

### Cancer-associated fibroblasts (CAFs)

Pan-cancer single-cell RNA-seq analysis of non-immune stromal cells from 16 cancer types and multi-platform spatial transcriptomics analyses of tumor tissues have recently been performed [29]. CAFs enriched at the tumor periphery interact with cancer cells to promote tumor progression and induce immune escape by functioning as a barrier to immune cell infiltration and modulating exhausted CD8^+^ T-cells [29]. Same-cell 10× Genomics Visium spatial transcriptomics, proteomics, and chromatin accessibility studies identify four main sub-types of CAFs in tumors, and suppressing the activity of one of the sub-types alters CAF cell spatial distributions and tumor progression [30, 31] (Fig. 2B).

10× Genomics Visium spatial transcriptomics profiling of colorectal cancer tissues uncovers that patients with primary tumors enriched of *F3*^+^ CAFs which secrete high levels of pro-tumor factors, such as *VEGFA*, *HGF*, *GDF15* and *NRG1*, are associated with poor prognosis [32]. Analyses of early-stage bladder cancer tissues identify PDGFRα^+^ITGA11^+^ CAFs that promote lymph angiogenesis and tumor invasion and are associated with poor patient prognosis [33].

NanoString GeoMx spatial transcriptomics analysis of non-small cell lung cancer tissues reveals that CAFs are linked with CD8^+^ T cell exhaustion, increased CD4^+^ T cell infiltration and immune exclusion [34]. 10× Genomics Visium analysis of glioblastomas shows that CAFs induce M2 macrophage polarization and glioblastoma stem cell enrichment through HGF and osteopontin [35], and that M2 macrophages secrete GPNMB to promote cancer cell transition into the mesenchymal subtype to enhance invasion [36] (Fig. 2B).

Single-cell and NanoString GeoMx spatial transcriptomics analyses of liver cancer tissues show that POSTN^+^ CAFs co-localize and interact with macrophages, leading to oncofetal reprogramming, epithelial-mesenchymal transition, tumor progression and relapse [37] (Fig. 2B), and high levels of CAFs in tumors are associated with poor patient prognosis [38]. 10× Genomics Visium profiling of breast cancer tissues reveal spatial organization of CAF-related niches. The TGFβ inhibitory gene *EMILIN1* is over-expressed in IFNγ-inflammatory CAF cells and is associated with high levels of CD8^+^ T-cell infiltration and better clinical outcomes [39] (Fig. 2B). In addition, 10× Genomics Visium analysis shows that FAP^+^ inflammatory and myofibroblastic CAFs are differentially localized in breast cancer tumors, and breast cancer cells induce detoxification-associated inflammatory CAFs to transit to immunosuppressive extracellular matrix (ECM)-producing myofibroblastic CAFs [40]. These data are not contradictory but show that different subtypes of inflammatory CAFs can promote or suppress immune responses, depending on their specific molecular characteristics and interactions within the TME.

In summary, spatial transcriptomics analysis reveals that CAFs interact with cancer cells, cancer stem cells, and immune cells to promote immune escape, tumor progression and metastasis, while inflammatory CAFs can promote or suppress immune responses.

### Lymphocytes

Single-cell and spatial transcriptomics profiling of pancreatic ductal adenocarcinoma tissues reveals exhausted CD8^+^ T cells, CD4^+^ T cells and NK cells, along with immunosuppressive myeloid cells [41]. Analyses of high-grade serous ovarian cancer tissues show that the cancer cell malignant state predicts NK cell and T cell infiltration and immune checkpoint blockade response [42].

Single-cell and spatial transcriptomics profiling of glioblastomas shows that IL10-secreting HMOX1^+^ myeloid cells, at mesenchymal-like regions, cause T cell exhaustion and thereby immune suppression [43]. Analysis of lymph nodes from B cell non-Hodgkin lymphoma patients shows that significant variations in IKZF3^+^ regulatory T cells, T follicular helper cells, or PD1^+^ TCF7^-^ cytotoxic T cells are associated with clonal expansion; and that high levels of PD1^+^ TCF7^-^ cytotoxic T cells correlate with poor patient outcomes [44].

Single-cell and spatial transcriptomics profiling reveals a widespread transition from B cells to plasma cells and the presence of dysfunctional TOX⁺CD8⁺ T cells in melanoma brain metastasis tumors [45], as well as a considerable clonal overlap between proliferating and exhausted CD8^+^ T cells, but little overlap with other tumor-infiltrating or circulating CD8^+^ T cells in brain metastases from melanoma, lung or breast cancer [46].

Tertiary lymphoid structures (TLSs) are organized immune cell clusters. Stereo-seq and 10× Genomics Visium spatial transcriptomics profiling of renal cell and nasopharyngeal carcinoma tissues reveals that CXCL13^+^ CAFs in TLSs activate exhausted CD8^+^ T cells, converting them into effector T cells, and induce B cell maturation and antibody generation within TLSs [47, 48] (Fig. 2C), suggesting that CAFs can play roles in tumor promotion and suppression under different TMEs. Spatial transcriptomics analyses of gastric cancer tissues show that immature TLSs in peritoneal metastases activate pro-tumor pathways, such as T cell exhaustion, while mature TLSs in primary gastric cancer tissues demonstrate anti-tumor immune responses [49] (Fig. 2C).

In summary, lymphocytes, particularly exhausted CD8^+^ T cells, play a central role in immune evasion. Mature TLSs induce B cell maturation and antibody generation and activate exhausted CD8^+^ T cells, while immature TLSs exacerbate immune suppression.

## Spatial transcriptomics enables the evaluation of tumorigenesis

Spatial transcriptomics allows for the high-resolution mapping of gene expression within tissue samples, providing insights into the molecular mechanisms underlying tumorigenesis. High-grade serous ovarian carcinomas, pre-cancerous serous tubal intraepithelial carcinomas, and counterpart normal tissues have been examined with spatial transcriptomics. Persistent interferon signaling, chromosomal instability, dysregulated innate and adaptive immunity, and IGFBP2 up-regulation have been discovered in pre-cancer lesions; and IGFBP2 induces epithelial cell proliferation and tumorigenesis [50, 51] (Table 1). Similar analyses of normal, pre-cancerous dysplasia, early-stage oral or esophageal squamous cell carcinoma tissues reveal substantial ANXA1 down-regulation during malignant transformation and progression, aberrant epithelial gene signature, disruption of the epithelial-stromal interface, fibroblast cell transformation to CAFs, the formation of CAF and epithelial cell niches, as well as immune-suppressive monocytes, collectively promoting tumor initiation [52]. Spatial transcriptomics analysis of genome-wide copy number variations in paired benign and cancerous prostate tissues uncovers unique clonal patterns in both tumors and neighboring benign tissues, suggesting that genomic instability in benign tissues is an early event in tumorigenesis [53] (Table 1).

**Table 1:** Spatial transcriptomics enables large-scale evaluation of tumorigenesis.

Cancer type	Tissues examined	Cause of tumorigenesis	References
Ovarian cancer	Normal, serous tubal intraepithelial carcinoma & ovarian carcinoma tissues	Persistent interferon signaling, chromosomal instability, dysregulated innate and adaptive immunity, and IGFBP2 up-regulation are discovered in pre-cancer lesions; and IGFBP2 induces epithelial cell proliferation.	[50, 51]
Oral and esophageal squamous cell carcinoma	Normal, pre-cancerous dysplasia, oral or esophageal squamous cell carcinoma tissues	ANXA1 down-regulation, aberrant epithelial gene signature, fibroblast cell transformation to CAFs, epithelial-stromal interface disruption, and immune-suppressive monocytes.	[52]
Prostate cancer	Benign and malignant prostate tissues	Unique clonal patterns in tumors and benign tissues suggest that copy number variations in benign tissues are an early event in tumorigenesis	[53]
Glioblastoma	A mouse model of glioblastoma	Biglycan is up-regulated in tumor-initiating cells, activates Wnt/β-catenin pathway, and promotes the mesenchymal sub-type and brain tumor-initiating cell proliferation and tumor initiation.	[54]
Wilms tumors	Wilms tumor and normal kidney tissues	Nephrogenic progenitor cells over-express SIX2 and CITED1 and exhibit cancer stem cell characteristics. ITGβ4 and ITGβ1 integrins regulate the self-renewal of SIX2 and CITED1 over-expressing nephrogenic progenitor cells.	[55]
Esophageal squamous cell carcinoma	Pre-cancerous and multistage esophageal squamous cell carcinoma tissues from mice and patients	TAGLN2 and CRNN are up- and down-regulated respectively when pre-cancerous lesions develop into carcinomas. Aberrant epithelial cell interactions in pre-cancerous lesions trigger epithelial-mesenchymal transition and cell cycle progression during tumor initiation & progression.	[57]
Pancreatic ductal adeno-carcinoma	Low or high-grade intraductal papillary mucinous neoplasms and related cancer tissues	ZNF117/HOXB3 as markers for low-grade dysplasia, SPDEF/gastric neck cell markers for borderline lesions, and NKX6-2/gastric isthmus cell markers for high-grade dysplasia neoplasm; and TNFα/Myc activation and polyamine enzyme in malignant initiation and further progression.	[58, 59]
Gastric cancer	Intestinal metaplasia	Intestinal metaplasia heterogeneity, including an intestinal stem-cell dominant cellular compartment, is associated with tumorigenesis. A novel clinical-genomic combination model outperforms clinical-only model in predicting intestinal metaplasia malignant transformation.	[60]
Cortisol-producing adenoma	Adrenal cortex adjacent to cortisol-producing adenomas	Zona reticularis layer displays macrophage-mediated immune response, and zona fasciculata expansion and consequent cortisol-producing nodules are associated with adenoma formation.	[103]
Gallbladder adeno-carcinoma	Biliary intraepithelial neoplasia, gallbladder adenocarcinomas and normal epithelium	Limited intra-patient transcriptomic variations between precursors and cancer lesions. Low-grade preneoplastic lesions can efficiently induce cancer, and SEMA4A promotes tumorigenesis.	[61]
Colorectal cancer	Colorectal adenomas, carcinomas and advanced cancer	Adenoma, Treg and cancer cell co-localization and interaction are important for tumorigenesis and mediated by midkine produced by tumor cells.	[62]

Spatial transcriptomics and single-cell RNA-seq studies of mouse glioblastoma tissues demonstrate considerable up-regulation of ECM factors, such as biglycan, which activates Wnt/β-catenin pathway, promotes mesenchymal phenotypes, and induces brain tumor-initiating cell proliferation and tumor initiation [54]. Similar profiling of Wilms tumors uncovers that nephrogenic progenitor cells in tumor tissues express high levels of SIX2 and CITED1 and exhibit cancer stem cell characteristics [55] (Table 1).

Spatial transcriptomics analysis demonstrates considerable up-regulation of TAGLN2 and down-regulation of CRNN when esophageal squamous pre-cancerous lesions develop into esophageal squamous cell carcinoma, and the gene expression alterations continue when carcinoma further progresses [56]. Analysis of multistage esophageal squamous-cell carcinoma tissues from mice and patients demonstrates that aberrant epithelial cell interactions via EFNB1–EPHB4 trigger epithelial–mesenchymal transition and SRC/ERK/AKT-signaling-mediated cell cycle progression at the basal layer in precancerous lesions, and that these changes expand to the entire epithelial layer during tumor initiation and progression [57] (Table 1).

Spatial transcriptomics analysis of human pancreatic intraductal papillary mucinous neoplasm, the cancer precursor, and cancer tissues defines ZNF117 and HOXB3 as markers for low-grade dysplasia, SPDEF and gastric neck cell markers for borderline lesions, and NKX6-2 and gastric isthmus cell markers in high-grade dysplasia neoplasm; and demonstrates TNFα and Myc activation as well as polyamine-metabolizing enzyme up-regulation in malignant initiation and further progression [58, 59] (Table 1).

Single-cell and spatial transcriptomics profiling of intestinal metaplasia, a pre-cancer condition of gastric cancer, identifies metaplasia driver genes, including the chromatin regulator ARID1A and intestinal homeostasis factor SOX9. Importantly, a novel clinical-genomic combination model outperforms the clinical-only model in predicting intestinal metaplasia malignant transformation [60]. The findings offer opportunities for gastric cancer prevention.

Spatial transcriptomics analysis of biliary intraepithelial neoplasia, gallbladder adenocarcinoma and normal epithelium regions has revealed that there are limited intra-patient transcriptomic variations between precursor and cancer lesions, that low-grade pre-neoplastic lesions can efficiently induce cancer, and that SEMA4A promotes tumorigenesis [61]. In addition, spatial transcriptomics analysis of colorectal adenomas, carcinomas and advanced cancer tissues reveals colocalization of regulatory T cells (Tregs) and colorectal cancer cells at the adenoma-carcinoma interface. Adenoma, Treg and cancer cell interactions are important for tumorigenesis and are mediated by midkine produced by tumor cells [62] (Table 1).

In summary, spatial transcriptomics is a powerful tool for investigating tumorigenesis, enabling high-resolution mapping of gene expression in normal, pre-cancerous, and cancerous tissues, and offering unprecedented insights into the molecular dynamics of tumorigenesis.

## Spatial transcriptomics identifies novel biomarkers for diagnosis, risk stratification, and prognosis

### Diagnosis

Gene expression signatures in tissues identified through spatial transcriptomics can serve as molecular markers for the differential diagnosis. The majority of breast ductal carcinoma in situ does not progress to invasive ductal carcinoma, but differential diagnosis is often challenging. Spatial transcriptomics analysis of non-malignant breast tissues, in situ or invasive ductal carcinomas, defines gene expression signatures for the three subtypes, and the gene expression signatures can be used to diagnose with an accuracy of 98% [63].

Plexiform neurofibromas are benign tumors, and a subset progresses to the deadly malignant peripheral nerve sheath tumors. Spatial transcriptomics studies reveal that malignant transformation from neurofibroma precursor lesions reduces the expression of antigen presentation and immune response genes, and that malignant peripheral nerve sheath tumor cells exhibit deregulated expression of cell survival and mitosis pathway genes [64]. These signatures serve as molecular diagnostic markers to identify neurofibromas at high risk of malignant transformation for better diagnosis and cancer prevention [64].

Combined hepatocellular-cholangiocarcinomas show mixed features of the two subtypes, resulting in difficulties in diagnosis and treatment. Spatial transcriptomics analysis of hepatocellular-cholangiocarcinomas shows that in situ gene expression of the tumors can predict intrahepatic cholangiocarcinoma which is associated with high cholangiocyte marker gene expression and low hepatocytic marker gene expression, and vice versa [65]. Spatial transcriptomics can therefore improve diagnosis and treatment decision-making.

### Risk stratification and prognosis

In triple-negative breast cancer, spatial profiling distinguishes an immunoreactive TME—marked by PD-L1 expression, CD8⁺ T cell infiltration, and favorable prognosis—from an immunosuppressive TME characterized by CD8⁺ T cell exclusion and B7-H4 expression, which correlates with poor outcomes [66]. In prostate cancer, *MSMB* is identified as the most downregulated gene in high-grade regions, alongside ten other genes predictive of cancer risk and therapeutic response [24].

Small cell lung cancer shows marked intra-tumoral heterogeneity (ITH), with spatial and bulk RNA-seq analyses identifying distinct gene signatures and immune features. The ITHtyper gene signature aids in patient risk stratification and guides personalized therapy [67].

In pancreatic ductal adenocarcinoma, spatial analysis uncovers recurrent gene signatures in both cancer cells and CAFs, including a neural-like progenitor cell signature linked to treatment resistance and poor prognosis [68]. In head and neck squamous cell carcinoma, high levels of TREM2⁺ multinucleated giant cells are associated with increased central memory CD4⁺ T cells, stronger anti-tumor responses, and improved outcomes [69]. In addition, profiling of bladder cancer tissues reveals that enrichment of Cadherin 12^high^ cancer cells predicts poor prognosis in patients after surgery and/or chemotherapy, but better response to immune checkpoint therapy [70].

In summary, spatial transcriptomics can be used for risk stratification and prognosis prediction across various cancers. These insights help identify key biomarkers and novel therapeutic targets for the development of personalized treatment strategies.

## Spatial transcriptomics identifies resistance factors to anticancer therapies

Tuberous sclerosis complex tumors exhibit mTORC1 hyperactivity. Single-cell and spatial transcriptomics reveal stem-like tumor cells linked to T cell dysfunction, immunosuppressive macrophages, and rapamycin resistance [71]. These stem-like tumor cells overexpress midkine, and its inhibition restores rapamycin sensitivity in vitro and in vivo, highlighting midkine as a potential therapeutic target and a key mediator of resistance [71] (Table 2).

**Table 2:** Spatial transcriptomics identifies factors important for anticancer therapy resistance.

Cancer type	Therapy	Cause of therapy resistance	References
Tumors due to tuberous sclerosis complex	Rapamycin	Stem-like tumor cells are associated with T cell dysfunction and immunosuppressive macrophages and are resistant to rapamycin. Midkine inhibition overcomes stem-like tumor cell resistance to rapamycin in vitro and in vivo.	[71]
Melanoma (primary)	Immuno-therapy	Immunotherapy-treated melanomas contain CD68^+^ macrophages, CD45^+^ leukocytes, and S100B^+^ tumor cells. Five and three of the S100B signature genes predict sensitivity and resistance to immunotherapy respectively.	[72]
Melanoma (primary)	Anti-PD-1 or anti-PD-1/anti-CTLA-4	Mesenchymal-type melanoma cells are enriched in immune checkpoint blockade therapy-resistant tumor tissues, and TCF4 serves as a driver of the mesenchymal-type signature.	[73]
Melanoma (metastastic)	Anti-PD-1 and anti-CTLA-4 therapies	B cell signatures in peri-tumor lymphoid predict better patient outcomes, MITF^+^SPARCL1^+^ and CENPF^+^ melanoma subclone formation and low T-cell infiltration are associated with innate resistance, and loss of memory state in T cells leads to acquired resistance.	[74]
Skin basal cell carcinoma	Immune checkpoint inhibitor	Macrophages and CAFs correlate with CD8^+^ T cell exclusion, immune suppression and immune checkpoint inhibitor resistance. Activin A regulates transcription in macrophages and CAFs and causes anti-PD-1 resistance.	[75]
Clear-cell renal cell carcinoma	Anti-PD-1 and anti-CTLA-4 therapy	In immunotherapy-resistant tumors, tissue repair, wound healing and immunoregulatory pathways are activated. Mesenchymal-like tumor cells and myofibroblastic CAFs are in close proximity, and correlate with therapy resistance.	[77, 78]
Non-small cell lung cancer	Immune checkpoint inhibitor	Tumors with high CD163^+^ macrophage infiltration over-express *CD27*, *ITGAM* and *CCL5* which drive macrophage infiltration-mediated anti-PD1/PD-L1 therapy resistance.	[79]
Small-cell lung cancer	Anti-PD-L1 & chemotherapy combination	IFNγ-driven immunity transcripts predict long-term patient survival, and hypoxia and glycolytic pathway activation predict resistance to combination treatment with anti-PD-L1 and chemotherapy (Carboplatin cisplatin/etoposide).	[81]
Gastric cancer	Regorafenib and anti-PD-L1 therapy	Macrophage infiltration is associated with resistance to combination therapy with the tyrosine kinase inhibitor regorafenib and the anti-PD-L1 antibody Avelumab.	[80]
Triple-negative breast cancer	Various chemotherapy agents	Therapy-resistant tumors display pro-angiogenesis signaling activation, enhanced metabolism, and over-expression of copy number-gained genes, epithelial-to-mesenchymal transition genes and the *ACTN1* gene.	[82, 83]
Gastro-intestinal cancer	Cabozantinib and anti-PD-L1	VEGF and MET signaling and extracellular matrix activation and co-existence of immune suppression and anti-cancer immune responses in non-responders.	[84]
Hepatocellular carcinoma	Cabozantinib & anti-PD-1	Non-responding tumors are characterized by immune-poor regions and cancer stem cell marker	[85]
Large B-cell lymphoma	CAR-T therapy	PD-1^+^ T cells are in close proximity to PD-1^+^ macrophages or tumor cells in patients who do not respond well to CD19-targeting CAR-T therapy.	[86]

Spatial transcriptomics of immunotherapy-treated primary melanoma identifies three main compartments: CD68⁺ macrophages, CD45⁺ leukocytes, and S100B⁺ tumor cells, with the S100B⁺ signature outperforming other known markers [72]. Non-responders to immune checkpoint blockade show enrichment of mesenchymal-type melanoma cells, driven by TCF4, a key mediator of resistance to both targeted and immunotherapies [73] (Table 2).

Single-cell and spatial multi-omics analyses have recently been performed with tumors from metastatic melanoma patients displaying response, innate or acquired resistance to immunotherapy [74]. B cell signatures in peri-tumor lymphoid aggregates are associated with better patient outcomes, immunotherapy results in MITF^+^SPARCL1^+^ and CENPF^+^ melanoma sub-clone formation and low T cell infiltration in innate resistant tumors, and loss of memory state in T cells leads to acquired immunotherapy resistance [74] (Table 2).

Spatial transcriptomics profiling of naïve and Cemiplimab-resistant skin basal cell carcinomas reveals macrophages and CAFs associated with CD8^+^ T cell exclusion, immune suppression, and resistance to immune checkpoint inhibitors, including Cemiplimab, in skin cancer patients [75]. Activin A regulates transcriptional reprogramming in macrophages and CAFs and causes ECM remodeling and therapy resistance [75], suggesting Activin A as a novel factor in immune checkpoint inhibitor resistance (Table 2).

Spatial transcriptomics analysis uncovers a hypoxia-mediated intercellular hub comprising cancer cells, ALCAM^high^ macrophages and exhausted CD8^+^ T cells at the periphery of tumors, such as breast and colorectal cancer tumors [76]. Inhibition of HIF-1ɑ suppresses ALCAM expression in macrophages and exhausted CD8^+^ T cells and potentiates the antitumor function of T cells to augment the efficacy of immunotherapies [76].

Spatial transcriptomics dataset from clear-cell renal cell carcinomas from patients treated with immune checkpoint blockade anti-PD-1 and anti-CTLA-4 agents has recently been analyzed [77]. In immunotherapy-resistant patients, tissue repair, wound healing and immunoregulatory pathways are activated, presenting important information for overcoming therapy resistance [77]. In addition, mesenchymal-like tumor cells and myofibroblastic CAFs are at close proximity at the tumor-normal tissue interface, and the enrichment of myofibroblastic CAFs correlates with immune checkpoint inhibitor therapy resistance [78].

Reduced CD163⁺ macrophage infiltration in non-small cell lung cancer is linked to better outcomes with immune checkpoint blockade [79]. Spatial transcriptomics reveals that high CD163⁺ macrophage levels correlate with anti-PD-1/PD-L1 resistance via CD27, ITGAM, and CCL5 upregulation [79]. In gastric cancer, similar resistance to regorafenib plus Avelumab is associated with macrophage infiltration, elevated M2 macrophages in the TME, and increased S100A10 expression in tumor cells [80].

Spatial transcriptomic analysis of small-cell lung cancer tissues from patients treated with chemoimmunotherapy (anti-PD-L1 and chemotherapy agents carboplatin, cisplatin and etoposide) reveals that high levels of IFNγ-driven immunity transcripts predict long-term survival, and that hypoxia and glycolytic pathway activation predict chemoimmunotherapy resistance [81].

Single-cell and spatial transcriptomics of triple-negative breast cancer reveal a population of chemo-resistant basal epithelial cells marked by copy number gains and EMT gene expression, both linked to poor prognosis. A chemo-resistance gene signature was identified, with ACTN1 emerging as a key driver and potential biomarker and therapeutic target [82]. Additionally, tumors from patients with complete chemotherapy response show lymphocyte infiltration and active IFN signaling, whereas resistant tumors display pro-angiogenic signaling and elevated metabolic activity [83].

A phase II trial of cabozantinib plus durvalumab in chemo-refractory gastrointestinal cancer patients revealed that non-responders, based on spatial transcriptomics of pretreatment tumors, exhibited VEGF/MET signaling, ECM activation, and immune exclusion—barriers that impair T cell–tumor interaction despite active immune responses. These findings underscore the value of spatial transcriptomics in guiding personalized therapy [84].

Spatial transcriptomics of hepatocellular carcinoma reveals that responders to cabozantinib plus nivolumab show immune- and CAF-rich tumors with pro-inflammatory signaling, including elevated B cell markers and maturation [85]. In contrast, non-responders exhibit immune-poor regions and cancer stem cell marker expression, suggesting new targets to overcome resistance [85].

Spatial transcriptomics of large B-cell lymphoma biopsies before CD19 CAR-T therapy show that responders have higher expression of T cell trafficking/function genes and fewer PD-L1⁺ macrophages and tumor cells [86]. In contrast, non-responders exhibit close proximity of PD-1⁺ T cells to PD-L1⁺ cells, implicating the PD-1/PD-L1 axis in CAR-T resistance [86] (Table 2).

In summary, spatial transcriptomics can be used to identify factors important for resistance to anticancer therapies, including chemotherapy, targeted therapy, immunotherapy, and combination therapy, as well as to help pinpoint novel therapeutic targets to overcome therapy resistance.

## Spatial transcriptomics data inform patient treatment decisions

**Fig. 3:**
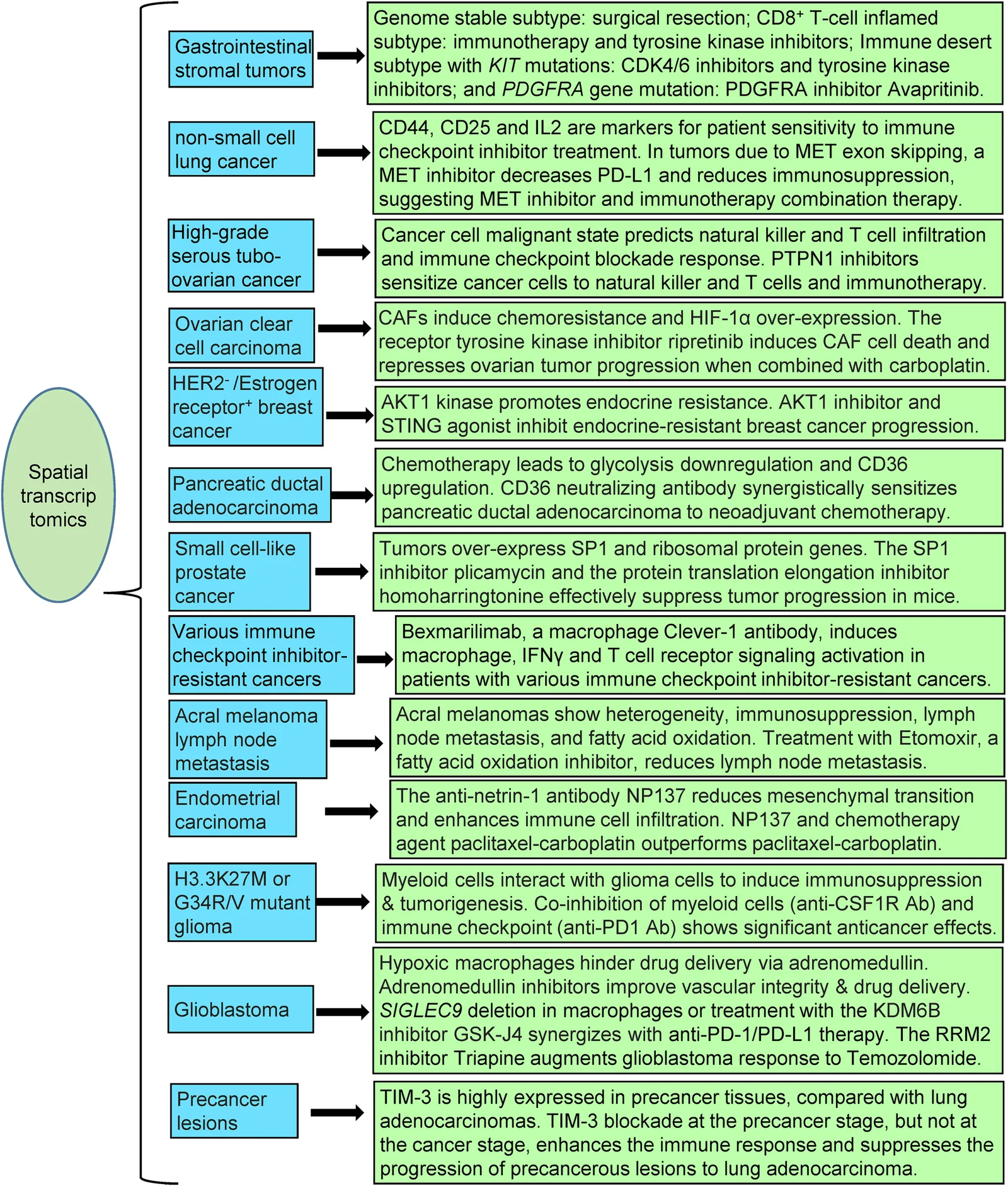
Spatial transcriptomics data inform cancer treatment and prevention decisions. Spatial transcriptomics analysis of tumors from patients, conducted before and/or after treatment with chemotherapy, immunotherapy or targeted therapy, identifies factors contributing to treatment sensitivity or resistance. Spatial transcriptomics data, therefore, help identify effective treatment options for various human cancers.

Spatial transcriptomics data can also be used to inform treatment decisions. Spatial transcriptomics of human gastrointestinal stromal tumors identifies four subtypes, each guiding subtype-specific treatments [87]. The genome-stable subtype requires only surgical resection; the CD8 + T cell-inflamed subtype may respond to immunotherapy combined with tyrosine kinase inhibitors; the immune-desert subtype, with KIT gene mutations, may benefit from CDK4/6 inhibitors and tyrosine kinase inhibitors; and the PDGFRA-mutant subtype should be treated with the PDGFRA inhibitor Avapritinib [87] (Fig. 3).

Digital spatial profiling shows that tumors from non-small cell lung cancer patients responsive to immune checkpoint inhibitors have CD44 depletion, reduced immuno-inhibitory marker expression, and overexpression of CD25 in tumors, along with IL2 overexpression in the stroma. These changes in CD44, CD25, and IL2 serve as biomarkers for guiding immune checkpoint inhibitor treatment in these patients [88] (Fig. 3).

Single-cell, spatial, and perturbational transcriptomics of high-grade serous tubo-ovarian cancer reveal that the malignant state of cancer cells predicts NK and T cell infiltration, as well as response to immune checkpoint blockade [42]. PTPN1 inhibition sensitizes ovarian cancer cells to T cell- and NK cell-mediated cytotoxicity, supporting combination therapy with PTPN1 inhibitors and immunotherapies [42] (Fig. 3). In ovarian clear cell carcinomas, chemoresistant tumor cells show HIF-1α overactivity in regions enriched with myofibroblastic CAFs, which induce chemoresistance via PDGF receptor signaling. Ripretinib induces CAF cell death and inhibits tumor progression when combined with carboplatin, offering an effective combination therapy against CAFs and ovarian clear cell carcinoma [89] (Fig. 3).

Bulk RNA-seq and spatial transcriptomics of estrogen receptor + /HER2- breast cancer tissues from patients post-endocrine therapy show reduced innate immune signaling and cytosolic DNA sensing in endocrine-resistant cells [90]. Combination therapy with an AKT1 inhibitor and STING agonist suppresses tumor progression in mice, offering a potential treatment for resistant cases [90]. In pancreatic ductal adenocarcinomas, neoadjuvant chemotherapy downregulates glycolysis and upregulates CD36, correlating with poor survival [91]. CD36 neutralizing antibodies enhance chemotherapy sensitivity, providing a promising approach for improved outcomes [91] (Fig. 3).

Spatial and single-nucleus transcriptomics of prostate cancer tissues identify a small cell-like carcinoma subtype, androgen receptor-positive and neuroendocrine-negative, with SP1 and ribosomal protein overexpression [92]. SP1 inhibitor Plicamycin and translation elongation inhibitor Homoharringtonine suppress tumor progression in mouse models, offering novel treatments for this subtype [92] (Fig. 3). In the first-in-human trial of Bexmarilimab, a macrophage Clever-1 monoclonal antibody, spatial transcriptomics shows that the therapy activates macrophages and IFNγ/T cell receptor signaling only in responsive patients with gastric, hepatocellular, breast, biliary tract cancers, and melanoma [93] (Fig. 3).

RNA-seq and spatial transcriptomics reveal significant tumor heterogeneity, an immunosuppressive microenvironment, and enhanced fatty acid oxidation in MYC+ acral melanomas, with strong ties to lymph node metastasis [94]. Treatment with Etomoxir, a fatty acid oxidation inhibitor, reduces metastasis, suggesting a therapeutic approach for these patients [94] (Fig. 3). In melanoma, high succinyl-CoA levels promote T cell-mediated tumor eradication through PD-L1 succinylation. The hyperlipidemia drug bezafibrate enhances the effects of CTLA-4 monoclonal antibodies to suppress melanoma progression [95].

Spatial transcriptomics of endometrial carcinoma biopsies show that the anti-netrin-1 antibody NP137 reduces EMT, boosts immune cell infiltration, and enhances cancer-microenvironment interactions. When combined with paclitaxel/carboplatin, NP137 outperforms chemotherapy alone, suggesting netrin-1 blockade sensitizes tumors to chemotherapies [96] (Fig. 3).

Single-cell, spatial transcriptomics, and proteomics of H3.3K27M/G34R/V mutant gliomas reveal myeloid cell infiltration enriched with immune checkpoint markers. Dual inhibition of myeloid cells (anti-CSF1R) and checkpoint pathways (anti-PD1) shows significant anticancer effects in mouse models, offering a new therapeutic strategy [97]. In glioblastomas, hypoxic monocyte-derived TAMs activate adrenomedullin signaling, impairing drug delivery. Adrenomedullin inhibitors improve vascular integrity and enhance treatment efficacy in vivo [98]. Monocyte-derived macrophages expressing SIGLEC9 are found in glioblastomas resistant to anti-PD-1 therapy. SIGLEC9 deletion activates T cells and synergizes with anti-PD-1/PD-L1 therapy, suggesting SIGLEC9 as a novel target to improve glioblastoma treatment [99] (Fig. 3).

Single-cell and spatial transcriptomics of glioblastoma reveal high KDM6B expression in immune-suppressive myeloid cells. Treatment with KDM6B inhibitor GSK-J4 activates pro-inflammatory signaling, reduces tumor-promoting myeloid cells, and enhances anti-PD1 efficacy in mice, suggesting a promising KDM6B inhibitor/anti-PD1 combination therapy [100] (Fig. 3). RRM2 overexpression and elevated dGTP levels are observed in glioblastomas post-temozolomide therapy. The RRM2 inhibitor Triapine boosts the response to temozolomide in mice, providing a new approach to overcome temozolomide resistance [101].

In lung adenocarcinoma mouse models, high TIM-3 expression is seen in precancerous lesions. TIM-3 blockade at the precancer stage enhances the immune response and prevents tumor progression, suggesting TIM-3 targeting as a strategy to stop precancerous lesions from progressing to cancer [102] (Fig. 3).

In summary, spatial transcriptomics data can be employed to guide precision cancer treatment and prevention.

## Conclusions and perspectives

As a transformative technology, spatial transcriptomics enables the mapping of gene expression within the spatial context of cancer tissues. By preserving the architectural organization of tumors, it provides insights into tumor sub-regions and compartments, such as TLSs, cellular composition and heterogeneity, inter- and intra-tumor variability, TME, immune responses, and cell-cell interactions. This approach surpasses traditional bulk or single-cell transcriptomics by uncovering crosstalk among cancer, immune, and stromal cells, as well as revealing how cell–cell interactions and spatial relationships regulate tumor initiation, progression, metastasis, and cancer cell responses to chemotherapy, immunotherapy, and targeted therapies. Furthermore, it facilitates the discovery of biomarkers for diagnosis and risk stratification and the prediction of effective therapies for individual patients. By unraveling the mechanisms of stepwise tumorigenesis from pre-cancerous lesions, spatial transcriptomics is paving the way for potential cancer prevention.

Spatial transcriptomics uncovers mechanisms of immune evasion or activation within the TME, offering critical information for the development of next-generation immunotherapies. Future directions in this field include integrating spatial transcriptomics with multi-omics technologies, such as proteomics, epigenomics, and metabolomics. This integration will enable a comprehensive understanding of tumor biology by linking gene expression with protein localization, expression, and metabolic activity within the tumor. These developments will significantly enhance our understanding of tumor initiation, progression, metastasis, immune response, cell-cell interactions, diagnosis, risk stratification, and therapy response or resistance.

A significant area of growth lies in the clinical application of spatial transcriptomics. In future research, combining spatial transcriptomics with artificial intelligence and machine learning can enable researchers and clinicians to identify biomarkers for prognosis, risk stratification, and therapeutic response prediction. Longitudinal studies, tracking spatial and temporal features of tumors before, during and after treatments, will aid in understanding how cancer evolves and adapts, will provide key information on how to predict patient response and therefore how to select the right treatment for individual patients, and will help design combination therapies to overcome cancer cell resistance. With innovations in cost-effective and scalable platforms, spatial transcriptomics has the potential to become a routine tool for personalized medicine.

Finally, spatial transcriptomics will broaden its impact by addressing diverse patient populations, rare cancers and cancer subtypes. Applying this technology to rare cancers or subtypes, such as rare pediatric cancers, or incorporating it into 3D models and patient-derived organoids, can offer new insights into tumor initiation, progression, risk stratification, and therapy response. Functional studies utilizing tools, such as CRISPR gene knockdown or knockout, single-cell perturbation assays, and experimental therapies in mouse models will validate spatial findings. As spatial transcriptomics continues to evolve, it will become a cornerstone of cancer research, bridging basic biology with clinical applications to drive innovative advancements in diagnosis, prognosis, and treatment.
